# Therapeutic credibility in community-based acupuncture care: an ethnographic study in southwest China

**DOI:** 10.3389/fsoc.2026.1908107

**Published:** 2026-08-26

**Authors:** Xu Xiao, Shariffah Suraya Syed Jamaludin, Yulian Cheng

**Affiliations:** 1 School of Social Sciences, Universiti Sains Malaysia, Minden Heights, Malaysia; 2 School of Literature and Media, Baise University, Baise, Guangxi, China

**Keywords:** acupuncture, China, ethnography, lay health knowledge, medical pluralism, relational trust, therapeutic credibility, Traditional Chinese Medicine

## Abstract

**Introduction:**

Community-based acupuncture care provides a useful setting for examining how patients judge treatment within plural healthcare systems.

**Methods:**

Drawing on six months of ethnographic fieldwork in Baise, Guangxi, including more than 300 h of participant observation and 29 in-depth interviews with 17 participants (11 patients, 10 of whom were women, and 6 practitioners), this study examines how patients decide whether acupuncture is plausible, safe, and worth continuing. The study focuses on patient-reported improvement; standardized clinical outcome measures were not collected. Lay therapeutic evidence refers here to the bodily, temporal, practical, social, and relational grounds patients use in making these judgments.

**Results:**

Patients compared bodily change over time, weighed treatment against work, family responsibilities, cost, and medication burden, consulted interpersonal and digital sources, and assessed practitioner explanation, attentiveness, visible safety practices, and referral decisions. Their judgments remained provisional: relief could coexist with doubt, trust with continued biomedical monitoring, and recommendation with hesitation.

**Discussion:**

The study conceptualizes therapeutic credibility as an ongoing and revisable judgment assembled from these everyday evaluations. It contributes to medical sociology by showing how patient-reported improvement becomes consequential in plural care through continuing comparison, interpretation, and revision.

## 1 Introduction

In everyday therapeutic life, patients do not judge acupuncture through clinical indicators alone. They also attend to pain, sleep, mobility, recurrence, bodily comfort, medication burden, and their ability to continue working or caring for family members (Kleinman, 1980; Csordas, 1990). Sensations such as soreness, numbness, warmth, and deqi may acquire meaning through previous treatment, daily routines, practitioner explanation, and social comparison (Farquhar, 1994; Pritzker, 2011). Patient-reported improvement may overlap with changes captured by clinical measures, but the present study does not estimate the degree of correspondence. Its focus is on how patients interpret perceived benefit and how it becomes consequential in decisions to continue, combine, or reconsider care.

This question is particularly relevant in contemporary China, where biomedical hospitals, state-recognized Traditional Chinese Medicine (TCM), lineage-based healing, community practice, household remedies, wellness services, and digital health information coexist (Zhan, 2009; Yu, 2021). Patients often move among these forms of care rather than treating them as mutually exclusive. Plural healthcare therefore involves continuing comparison, monitoring, and revision rather than a single choice between medical systems.

Baise, Guangxi, provides a distinctive setting for examining this process. This mountainous borderland city brings biomedical institutions, state-recognized TCM, Zhuang and other local medical knowledge, pharmacies, household remedies, wellness services, community practitioners, and platform-based information into close interaction. Its combination of formal and community-based resources makes visible how patients assess care where institutional authority, local reputation, bodily experience, and therapeutic explanation intersect.

Research on explanatory models, medical pluralism, embodiment, patient knowledge, and healthcare trust has established that treatment is interpreted through situated meanings and relationships rather than through fixed medical logics alone (Kleinman, 1980; Farquhar, 1994; Csordas, 1990; Pols, 2014; Yu, 2021). However, existing studies have often concentrated on why patients initially seek complementary or traditional care, or have examined bodily experience, social validation, digital information, and practitioner authority as separate issues. Less is known about what happens after patients enter community-based acupuncture care. Continued treatment requires them to monitor relief and recurrence, compare acupuncture with previous care, negotiate work and family responsibilities, consult interpersonal and digital sources, and judge practitioner responsibility and safety across repeated encounters.

To clarify the analytic sequence, this study distinguishes four closely related terms. Perceived benefit refers to patient-reported improvement as described by participants. Lay therapeutic evidence refers to the bodily, temporal, practical, social, and relational grounds patients use to judge whether treatment is plausible, safe, and worth continuing. Therapeutic evaluation refers to the ongoing process through which these grounds are compared, tested, and revised. Therapeutic credibility refers to the provisional judgment that emerges from this process and informs whether care is continued, combined with other treatment, or reconsidered. Perceived benefit is therefore one component of lay therapeutic evidence rather than a synonym for therapeutic credibility.

Drawing on 6 months of ethnographic fieldwork in four community-based acupuncture settings in Baise, this study examines how patients construct, test, and revise therapeutic credibility. It asks:

RQ1: How do patients evaluate community-based acupuncture in relation to prior biomedical treatments, and how do they interpret and test perceived bodily change over time?

RQ2: How do patients negotiate treatment decisions under practical constraints and draw on community and digital networks to validate their evaluations?

RQ3: How do practitioner explanations, visible safety practices, and recognition of therapeutic limits shape relational trust and the continued credibility of acupuncture care?

By tracing how bodily experience, temporal comparison, practical constraints, social and digital information, and practitioner conduct interact across continued treatment, this article extends research on medical pluralism beyond initial care-seeking. It offers a grounded account of how therapeutic credibility is assembled and revised where institutional guarantees, local recognition, and regulatory visibility vary. The following section reviews the literature relevant to these processes before describing the ethnographic design and findings.

## 2 Literature review

### 2.1 Comparing treatments in plural care

Patients living within plural healthcare systems rarely rely on a single form of care. They move among hospitals, Traditional Chinese Medicine (TCM), community practitioners, household remedies, and advice from relatives or acquaintances while deciding what remains worth pursuing. These movements involve continuing comparisons of explanations, previous outcomes, treatment burdens, and the practical demands of everyday life.

Work on explanatory models and medical rationality shifted attention from medicine as fixed professional knowledge to the situated meanings through which illness and treatment become intelligible (Kleinman, 1980; Good, 1994). Research on Chinese medicine develops this insight by showing that biomedical and traditional care do not necessarily operate as mutually exclusive systems. Chinese medicine is internally plural and continually shaped through clinical interaction, institutional change, local knowledge, and transnational relations (Farquhar, 1994; Scheid, 2002; Zhan, 2009). In southwest China, biomedical and local therapeutic practices similarly coexist through compatibility, competition, and everyday negotiation (Yu, 2021).

Patients may therefore use hospitals for diagnosis, imaging, surgery, acute illness, or serious disease while seeking community-based care for chronic pain, sleep problems, fatigue, rehabilitation, or bodily regulation. Survey research in China also indicates that preferences for TCM vary by medical condition and are particularly associated with prevention, chronic illness, common symptoms, rehabilitation, and sleep-related concerns (Zhao et al., 2023). Such movement is better understood as practical comparison than as cultural loyalty to one medical tradition.

Much research on medical pluralism has focused on treatment entry, including how access, cost, beliefs, or dissatisfaction shape initial attendance. Less attention has been paid to what happens after treatment begins. Continued care requires patients to compare relief with recurrence, judge whether treatment fits work and family routines, interpret practitioner explanations, and consider risks associated with delayed formal assessment. Entering acupuncture therefore begins, rather than ends, a continuing process of therapeutic evaluation.

### 2.2 Interpreting bodily change over time

Once patients enter treatment, the question is not only which therapy to choose, but how to decide whether change is meaningful. Community-based acupuncture often requires patients to interpret gradual and ambiguous experiences. Relief may be immediate or delayed, sensations may reassure or unsettle, and improvement may coexist with uncertainty.

The embodiment perspective provides a starting point for understanding this process. (Csordas 1990) treats the body not as a passive object of intervention, but as the ground through which people perceive and understand the world. In Chinese medicine, soreness, numbness, warmth, heaviness, fatigue, changes in sleep, and shifts in vitality acquire meaning through therapeutic language and interaction rather than sensation alone (Farquhar, 1994; Scheid, 2002; Pritzker, 2011). The same needling response may be interpreted as deqi, therapeutic activation, excessive stimulation, or possible injury.

Therapeutic judgment is also temporal. Patients compare present experience with remembered bodily states, monitor recurrence, and assess whether improvement persists beyond the treatment encounter. What matters is not simply whether a symptom changes, but whether the change affects work, sleep, movement, medication use, or family responsibilities. Bodily experience becomes persuasive through repeated comparison rather than through immediate sensation alone.

Research on patient knowledge further complicates a simple division between professional expertise and lay belief. People living with illness develop practical knowledge through repeated encounters with symptoms, treatment, and everyday adaptation (Pols, 2014), while formal evidence systems may not fully capture such situated knowledge (Adams, 2016). Patient judgments may sometimes converge with changes captured by clinical measures and at other times diverge from them; in both cases, they shape treatment decisions.

As defined in the Introduction, lay therapeutic evidence comprises the practical grounds through which patients judge treatment to be credible enough to continue. The concept names how patients reason with bodily, temporal, practical, social, and relational information under uncertainty; it does not presume either concordance or conflict with formal outcome assessment. Relief may coexist with doubt, and explanation may clarify a sensation without fully resolving uncertainty.

### 2.3 Therapeutic agency under constraint

Interpreting bodily change does not automatically determine what patients do next. Even when treatment appears beneficial, decisions to continue, combine, postpone, or discontinue care remain shaped by circumstances beyond bodily experience. This evaluative process is therefore inseparable from the conditions under which patients are able to act.

Research on chronic illness challenges the assumption that patients simply comply with or reject professional advice. Long-term illness disrupts everyday life and requires continuing adjustments to routines, relationships, and expectations (Bury, 1982; Charmaz, 1983). (Mol 2008) similarly contrasts isolated choice with the ongoing adaptation involved in care. Burden of Treatment Theory argues that patients' capacity to manage illness depends on the work required by treatment and the resources available to carry it out (May et al., 2014).

These perspectives matter when recommendations are difficult to reconcile with daily responsibilities. Advice to rest, attend repeated appointments, tolerate side effects, or undergo further procedures may be difficult for patients responsible for wage labor or family care. Treatment decisions therefore reflect both perceived benefit and the demands of sustaining care.

Therapeutic agency in this context is not unrestricted autonomy, resistance to professional authority, or simple consumer choice. It is the situated capacity to compare options, combine recommendations, question particular advice, monitor consequences, and revise decisions while negotiating material, relational, and institutional constraints. Less is known about how this agency develops after patients accumulate therapeutic experience and compare professional recommendations with lived outcomes. When uncertainty remains, they may also turn to other people and digital sources for comparison.

### 2.4 Collective and digital validation

When bodily change remains uncertain, patients rarely rely on personal experience alone. Improvement may be incomplete, symptoms may fluctuate, and changes may be difficult to attribute to acupuncture rather than natural recovery or other care. Under these conditions, patients' evaluations become socially distributed.

Recommendations from relatives, friends, neighbors, and trusted acquaintances can reduce uncertainty by making unfamiliar treatment more intelligible and less risky. Such testimony does more than encourage initial attendance. It can shape how later bodily experiences are interpreted, because patients compare their responses with visible recovery and stories circulating through familiar relationships. Healthcare workers or medically informed acquaintances may be especially influential because their recommendations combine interpersonal familiarity with proximity to formal medical authority.

Digital platforms expand the range of experiences available for comparison. Meituan, WeChat, Xiaohongshu, and map-based services can make prices, locations, ratings, practitioner information, and accounts of bodily response searchable. Research on Chinese digital health communication suggests that users compare credibility signals and assess source reliability rather than passively accepting online information (Chang et al., 2024; Deng et al., 2019). Health literacy research likewise emphasizes the ability to access, interpret, and evaluate competing information (Nutbeam, 2000; Bains and Egede, 2011; Sørensen et al., 2012; Okawa et al., 2023), while digital health literacy remains unevenly distributed (Zhang et al., 2025).

Online information rarely functions as an independent source of authority in community care. Platform accounts become persuasive when they align with familiar recommendations, local reputation, observations in treatment settings, practitioner explanations, and later bodily experience. Agreement across sources may reduce uncertainty, whereas inconsistency may introduce doubt. The same process can amplify selective success stories, commercial promotion, or premature expectations. Less attention has been paid to how patients compare these sources after treatment begins and how these comparisons shape continued relationships with practitioners.

### 2.5 Relational trust with safety boundaries

Comparing bodily experience with social and digital information may reduce uncertainty, but it does not by itself sustain care. Continued treatment also depends on whether patients judge practitioners to be sufficiently trustworthy, responsible, and safe.

Research on healthcare trust treats trust as a relational and institutional achievement shaped by competence, communication, care, vulnerability, and perceived responsibility (Mechanic and Meyer, 2000; Hall et al., 2001; Gilson, 2003). In hospitals, authority is supported by licensing, diagnostic technologies, and organizational oversight. In community-based acupuncture, these guarantees may be uneven, so patients may rely more on local reputation, perceived skill, explanation, and moral restraint.

Practitioner explanations matter because bodily sensations do not carry fixed meanings. Linking symptoms with work, stress, diet, sleep, or bodily constitution can make treatment feel coherent. Yet familiarity and reputation may also discourage questions about technique, credentials, claims, or biomedical assessment. Adverse events associated with improper technique, hygiene problems, or inappropriate practice make these concerns consequential (Zhang et al., 2010).

Patients may judge responsibility through disposable needles, cleaning practices, respectful communication, acknowledgment of limits, and referral when symptoms require formal assessment. Such signs may support trust, but they are not equivalent to regulatory or infection-control oversight. Policy discussions accordingly emphasize quality assurance, practitioner regulation, appropriate use, and scope-of-practice boundaries (World Health Organization, 2025; Hu, 2021).

Existing scholarship shows that patients compare care, interpret bodily change, act within constraints, consult social and digital sources, and assess practitioner responsibility. These processes are often studied separately. Less is known about how they interact after patients enter community-based acupuncture care, or how perceived benefit becomes credible enough to guide treatment while remaining open to doubt and revision. This study addresses that gap through an ethnographic analysis of therapeutic credibility in Baise.

## 3 Materials and methods

### 3.1 Study design and sociological orientation

This study used interpretive ethnography to examine how patients constructed, tested, and revised therapeutic credibility in community-based acupuncture care (Hammersley and Atkinson, 2019). Therapeutic credibility was treated as a situated judgment open to revision, shaped by bodily sensations, temporal comparison, social testimony, practitioner explanation, digital checking, and visible safety or referral cues.

Ethnography enabled comparison between participants' accounts and repeated interactions in treatment rooms, waiting areas, informal conversations, and digital exchanges. The unit of analysis was the broader process of therapeutic evaluation through which bodily, temporal, practical, social, and relational grounds were compared and revised, allowing care to become credible, uncertain, or no longer worth continuing. The theoretical framework sensitized the analysis to comparisons across forms of care, bodily change over time, and socially organized credibility without predetermining the coding structure.

### 3.2 Research setting and case boundaries

Fieldwork was conducted in Baise City, Guangxi Zhuang Autonomous Region, southwest China. The setting was selected because biomedical hospitals, state-recognized Traditional Chinese Medicine, community practitioners, household remedies, wellness services, and digital health information coexist within a mountainous borderland context.

Community-based acupuncture refers here to acupuncture delivered alongside related practices, including moxibustion, cupping, massage, and health-cultivation advice, outside formal hospital departments. The descriptors informal, semi-formal, and semi-clinical are used only to indicate organizational position, regulatory visibility, and proximity to formal institutions; they are descriptive rather than analytical categories and do not imply practitioner skill or local legitimacy. The case included patients with direct experience of these settings and practitioners who provided such care; the analytic focus was how treatment was interpreted, compared, and acted on in ongoing care.

### 3.3 Site selection and field access

Four sites were purposively selected to capture variation in formality, spatial organization, patient flow, and practitioner-patient interaction: a residential family-based clinic, a health-cultivation center near a Traditional Chinese Medicine hospital, a border-county rehabilitation space, and a community acupuncture center. Approximate observation time was 90, 80, 65, and 70 h, respectively. Full site characteristics are provided in Supplementary Table S1. Access was negotiated through community gatekeepers and participant referral. The researcher did not intervene in diagnosis, treatment, payment, referral, or decision-making, and access was treated as an ongoing ethical relationship.

### 3.4 Participants and recruitment

Purposive sampling recruited adults who had completed at least three acupuncture sessions at a selected site within the previous year, and practitioners with at least 5 years of community-based experience or sustained local recognition through apprenticeship, mentorship, lineage, or reputation. These criteria ensured that participants had sufficient experience to discuss continuing comparison, uncertainty, safety boundaries, and referral decisions.

The study included 17 participants, comprising 11 patients and 6 practitioners, and 29 interviews, including 16 patient and 13 practitioner interviews. Recruitment combined gatekeeper introduction, direct invitation, and snowball sampling. Ten of the 11 patient participants were women, a feature considered analytically relevant because several sites and recruitment networks involved intimate health, fatigue, chronic pain, and body-management concerns. Participant and practitioner characteristics are summarized in Supplementary Tables S2, S3. Data collection continued until later material added variation, ambiguity, and negative cases but no longer altered the five-dimensional analytical structure. This pragmatic judgment was reviewed during peer and supervisory debriefing.

### 3.5 Data collection

Data collection combined semi-structured interviews, participant observation, informal conversations, and fieldnotes between November 2025 and April 2026. Formal data collection began after ethical approval on 12 November 2025. Interviews lasted 45 to 90 mins, were conducted in Mandarin Chinese, audio-recorded with consent, transcribed verbatim, and anonymized. The guide covered treatment history, bodily sensations, perceived benefit or disappointment, continuation or discontinuation, recommendation sources, practitioner communication, safety, and trust.

More than 300 h of observation were calculated from fieldwork logs and excluded formal interviews, transcription, translation, and desk-based analysis. Observation covered treatment and waiting-area interactions, informal and digital exchanges, payment and appointment practices, symptom descriptions, reassurance or fear, and visible safety-related practices. Disposable needles, alcohol-based wiping, situational use of gloves or masks, flame exposure of some cupping or glass instruments, and cleaning of reusable tools were recorded as visible practices rather than evaluated as an infection-control audit.

### 3.6 Data management and translation

Transcripts, fieldnotes, and analytic memos were de-identified and stored securely. Published excerpts were translated by a primary translator and checked against the Chinese transcript or fieldnote context by another team member, with particular attention to culturally specific terms such as deqi, yangsheng, han or cold, qi, constitution or tizhi, meridians, and regulation. Wording was revised when literal translation risked flattening participants' meanings or implying biomedical equivalence. Disputed terms were checked against the original Chinese, although full back-translation was not conducted for every excerpt.

### 3.7 Analytical strategy and theme development

Data were analyzed using reflexive thematic analysis informed by interpretive ethnography (Braun and Clarke, 2006, 2021). Analysis began during fieldwork and moved iteratively through familiarization, initial coding, pattern development, theme review, definition of analytical dimensions, and interpretive writing. Initial codes remained close to interview and observational material, including relief and recurrence, medication burden, work constraints, social and digital checking, practitioner explanation, privacy, visible safety practices, and referral.

**Figure 1:**
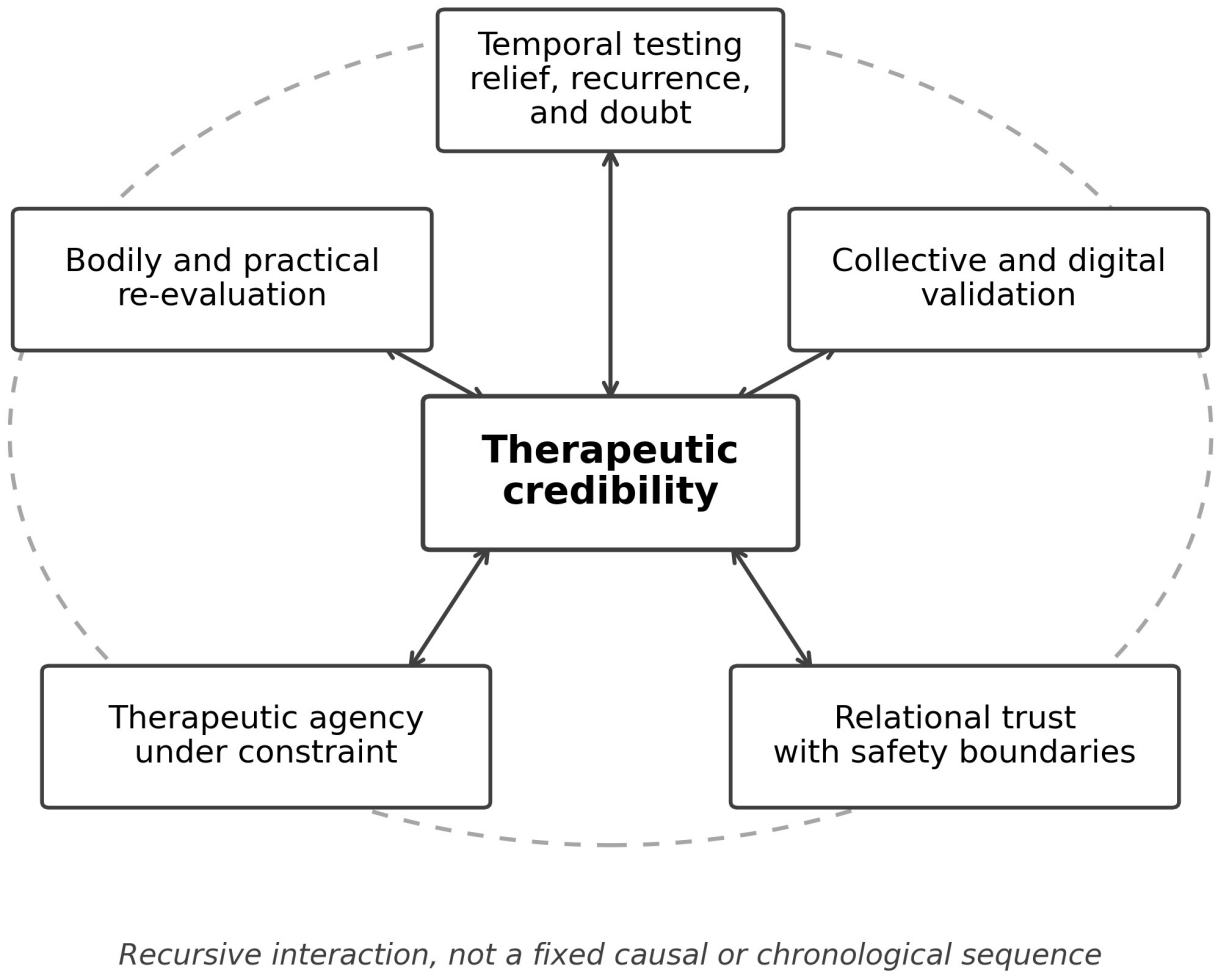
Recursive analytic model of therapeutic credibility in community-based acupuncture care. The dimensions interact recursively and do not represent fixed causal, chronological, or participant-level stages.

Focused coding developed five interrelated dimensions: bodily and practical re-evaluation, temporal testing, therapeutic agency under constraint, collective and digital validation, and relational trust with safety boundaries. Themes were retained when they accounted for both interviews and observed interactions, and were revised when they obscured ambiguity or negative cases. Table 1 summarizes theme development, and Figure 1 presents the recursive analytic model.

**Table 1:** Theme development from initial codes to analytical dimensions.

Analytical dimension	Initial codes	Theme development	Example evidence
Bodily and practical re-evaluation	Short-lived relief; medication burden; flexible access; tolerability; everyday fit	Effective care was judged through bodily response, treatment burden, accessibility, and everyday fit.	P1 compared medication with acupuncture; participants assessed proximity, scheduling, and session duration.
Temporal testing	Recurrence; duration; flare-up rhythm; technique differences; painful needling; doubt	Credibility was tested through duration, recurrence, later comparison, and doubt.	P4 compared flare-ups and practitioners; P1 initially interpreted strong needling as possible injury.
Therapeutic agency under constraint	Self-directed choice; professional advice; work, family, and cost constraints; delayed-care concern	Patients negotiated options within economic, familial, and medical limits.	P9 weighed advice against lived outcomes; P4 continued care while unable to rest.
Collective and digital validation	Word-of-mouth; medically informed endorsement; waiting-area comparison; Meituan and Xiaohongshu checking; convergence testing	Offline and digital sources made experiences socially interpretable and comparable; cross-source alignment was assessed through convergence testing.	Patients compared recommendations, platform accounts, observed interactions, and bodily responses.
Relational trust with safety boundaries	Feeling listened to; explanation; privacy violation; shaming; hygiene; referral; practitioner limits	Trust could strengthen through explanation, responsibility, visible safety, and referral, or weaken through disrespect and privacy failures.	P2 and P3 described explanatory trust; one participant reported a privacy-violating encounter; D2 referred; D6 acknowledged limits.

Initial codes are illustrative. The dimensions are analytical emphases rather than fixed stages or participant categories.

### 3.8 Trustworthiness, reflexivity, and transferability

Trustworthiness was supported through prolonged engagement across four sites, triangulation of interviews, observations, informal conversations, and analytic memos, an audit trail, and peer and supervisory debriefing. Debriefing involved the principal supervisor, who has expertise in medical anthropology, and a Baise-based university lecturer with expertise in local folk culture, and addressed theme boundaries, translation, alternative explanations, the gendered sample, and evidential grounding.

The primary researcher was trained in anthropology and sociology and had prior familiarity with Chinese medicine and community-based care. This familiarity and gatekeeper access aided rapport but could encourage positive interpretations of relational care or local legitimacy, so reflexive memos examined how disciplinary background, field relationships, and expectations shaped the analysis. Formal member checking was not used because the analysis was interpretive and synthesized accounts could compromise confidentiality in small, interconnected settings. Study trustworthiness instead relied on triangulation, negative cases, debriefing, an audit trail, and thick description; transferability rested on contextual detail rather than statistical generalization.

### 3.9 Ethical considerations

Ethical approval was obtained from an institutional human research ethics committee (committee name and approval code withheld for double-blind review; approval date: 12 November 2025). The study was conducted in accordance with the ethical principles of the Declaration of Helsinki (World Medical Association, 2025). All participants were adults and provided written informed consent for interviews. The researcher explained the study purpose, voluntary participation, possible discomfort, confidentiality, the absence of direct clinical benefit, and the right to withdraw without affecting care, payment, or relationships.

Consent for observation was ongoing. Practitioners consented to observation of their own clinical work, while patient-level consent was obtained for specific encounters. Patients could decline observation, ask that an interaction not be recorded, request that the researcher leave, or withdraw. Observation was avoided or stopped when discomfort or additional bodily privacy was evident. Data were de-identified, consent forms were stored separately, and no identifiable images or personal data are reported. Reporting distinguishes participants' accounts from the authors' interpretations and avoids endorsing any specific practitioner, treatment claim, or regulatory status.

## 4 Findings

The findings are organized around five interrelated analytical dimensions: bodily and practical re-evaluation, temporal testing, therapeutic agency under constraint, collective and digital validation, and relational trust with safety boundaries. The first two dimensions primarily address RQ1 by showing how patients compared acupuncture with prior care and tested bodily change over time. Therapeutic agency under constraint and collective and digital validation primarily address RQ2 by showing how patients negotiated decisions within practical limits and cross-checked their evaluations through community and digital networks. Relational trust with safety boundaries primarily addresses RQ3. Because the dimensions interact recursively, therapeutic agency under constraint also intersects with RQ1 and RQ3 when bodily evaluations are translated into decisions involving professional advice and perceived risk.

Although presented separately for analytical clarity, these dimensions were not fixed stages. Patients could compare acupuncture with medication, monitor bodily change, consult relatives or digital sources, and assess practitioner responsibility within the same treatment trajectory. They moved among these forms of evaluation recursively and sometimes ambivalently. The same cases therefore recur across sections when different moments in one trajectory illuminate more than one analytical dimension.

### 4.1 Bodily and practical re-evaluation

Patients redefined effective and livable care through bodily response, tolerability, medication burden, accessibility, and everyday fit rather than through symptom reduction alone. This section concerns the criteria through which care was evaluated; Section 4.3 examines how patients acted on those evaluations under practical constraints.

A recurrent finding across patient narratives was that dissatisfaction with biomedical care did not simply produce rejection of formal medicine. Instead, it prompted an active reassessment of what counted as effective care. Participants described this process through bodily experience, particularly when medication side effects, temporary relief, or unmet expectations made biomedical treatment appear limited or unsustainable. Patients' judgments were therefore grounded not only in formal authority but also in bodily response, tolerability, and practical livability.

P1's account illustrates this form of practical re-evaluation. Although biomedical treatment helped regulate clinical indicators, it did not resolve the discomfort that shaped her everyday life. She explained: “The medicated patches do work, but as soon as I take them off, the pain comes back. Oral painkillers are the same: they do not last long, and I am also afraid of damaging my body by taking too many.” For P1, side effects and repeated relapse became practical evidence of limitation. Treatment was judged not only by whether it produced temporary relief, but also by whether it restored bodily comfort in a sustainable way.

Practical evaluation also included whether symptoms and treatment could be managed within work and family routines. Because the decision-making consequences of these constraints are central to therapeutic agency, P4's treatment choice is examined in Section 4.3.

Practical livability also included temporal and spatial accessibility. One participant explained: “I can come here whenever I want; I just make an appointment on WeChat in advance. The timing is flexible, the effect is good, and it is also close to where I live. Each session takes about 40 mins, so it is very quick.” For this participant, proximity, flexible scheduling, and treatment duration formed part of the practical judgment of whether acupuncture could be incorporated into everyday routines and sustained over time.

Across these cases, patients re-evaluated treatment through bodily response, tolerability, medication burden, accessibility, everyday fit, and perceived risk. Bodily and practical re-evaluation therefore involved redefining effective care through its compatibility with everyday life rather than rejecting biomedical care as a whole. Yet these criteria did not settle the value of treatment at a single moment; they had to be tested over time.

### 4.2 Temporal testing: relief, recurrence, and doubt

Patients tested these revised standards through duration, recurrence, later comparison, and continuing doubt. They tracked the changing rhythm of symptoms across everyday life, using time to distinguish short-lived relief from bodily change that remained persuasive beyond the treatment session. Therapeutic credibility was therefore not immediately settled but repeatedly reassessed.

Temporal testing was also visible in observed encounters. After needling, patients sometimes rotated the neck, bent the waist, lifted an arm, walked, or pressed a painful area before commenting on change. Later reports of the return or persistence of pain helped distinguish an immediate bodily difference from improvement that remained persuasive beyond the session.

Returning to P1's account, she described her condition as a recurring occupational illness associated with prolonged office work: “This condition is basically an occupational illness. I work in an office and sit for 8 h a day. Sometimes when I get busy, I do not even get up and walk around, and over time, that causes problems. So it flares up two or three times a year. When it gets bad, it hurts even when I am lying down.” She contrasted the short duration of medicated patches and oral painkillers with acupuncture, which she said produced relief after the first session and more sustained improvement after about six sessions. What mattered was not simply that acupuncture reduced pain, but that improvement appeared to last in a way she had not experienced before.

P4's trajectory likewise shows how patients assessed treatment through long-term bodily observation. She recalled: “Previously, doctors also told me to take glucosamine and use medicated patches. To be honest, the effect was not obvious. The pain still came when it came, especially during acute flare-ups; it was unbearable. My knee would make cracking sounds, it would swell badly, and the fluid would still build up. Later, I tried acupuncture, especially once when I went during an acute flare-up. After the treatment, I felt much better. But this also depends on the technique. Different practitioners use different needling methods. I went to the Chinese medicine hospital before, and the effect there was not good. But this practitioner really could help.”

For P4, bodily change was interpreted by comparing the duration of relief, the recurrence of swelling and pain, and her ability to continue working across successive episodes. Temporal testing therefore extended her judgment beyond immediate relief to whether familiar symptom patterns changed across work and everyday life. Her confidence remained conditional on practitioner technique, the timing of treatment, and whether improvement persisted beyond an acute episode.

Yet temporal comparison could unsettle as well as strengthen therapeutic credibility. P1 described a strong needling technique that the practitioner called “waking the needle.” She felt an electric sensation spreading from her legs through her body and initially interpreted the pain as possible injury rather than therapeutic activation. She recalled: “To be honest, I felt that needling was a kind of injury. But the doctor said this meant the needle was in the right place, and that soreness, numbness, and distension meant that qi and blood were moving. I thought perhaps he had inserted it in the wrong place, into a tendon or meridian, which was why it cramped. But the doctor explained that this was a sign of improvement and of activating qi and blood. I was still a little doubtful.”

Across these cases, temporal testing kept therapeutic credibility unsettled. Bodily change became persuasive only insofar as it endured, altered recurrence, or remained meaningful after later comparison. Immediate sensation could strengthen confidence, but it could also generate fear and doubt. Improvement therefore did not close evaluation; it extended it. As these comparisons accumulated, they also shaped how some patients positioned themselves within treatment decisions.

### 4.3 Therapeutic agency under constraint

Over time, accumulated bodily comparisons became consequential for how patients negotiated treatment decisions under constraints of work, family responsibility, cost, professional advice, and perceived risk. Therapeutic agency concerned how patients acted on evaluative criteria under constrained conditions rather than unrestricted choice. For some participants, perceived improvement strengthened their confidence to compare treatment options, question particular recommendations, and make judgments on their own terms.

P9, a 40-year-old civil servant with recurrent headaches, described dissatisfaction with pharmaceutical treatment not because it offered no relief, but because its effects were limited and difficult to sustain. Medication could reduce individual attacks, yet it did not reduce their frequency and seemed to lose effectiveness with repeated use. Preventive treatment was also experienced as undesirable because of side effects and incompatibility with high-pressure administrative work. Under these circumstances, he began to explore acupuncture, moxibustion, and other therapeutic options more actively. He stated: “I listen to their professional advice, but I make the decisions, and I bear the consequences. I clearly know what works for me.”

This statement does not reject professional knowledge. Instead, it repositions the patient in relation to it. For P9, therapeutic choice was not framed as a refusal of medical authority but as selective and self-conscious judgment in which bodily experience became central to decision-making. The capacity to decide was grounded not in abstract resistance, but in repeated comparisons between professional recommendations and lived outcomes.

Other treatment decisions were similarly negotiated. Patients compared practitioner advice with hospital examinations, prescribed medication, family concerns, financial pressure, and the perceived risk of delayed formal care. Choosing acupuncture often meant combining recommendations while continuing to monitor bodily change.

Across the cases, patients often continued hospital examinations, prescribed medication, or other forms of care while trying acupuncture. Therapeutic agency therefore operated through comparison and adjustment rather than through replacement of one medical system by another.

P4's case shows how such constraints shaped treatment choice. A 27-year-old factory worker with long-term arthritis, she had repeatedly been advised to rest, manage symptoms, or consider surgery. In practice, rest was incompatible with wage-dependent labor and family responsibility. She stated: “I work in a chemical factory, and every day I am on the assembly line, standing for 8 to 12 h. My family depends on me alone, so I simply cannot stop working. The doctor said that the only way to manage this chronic condition was to stand less and rest more, and if it became more serious, then surgery would be the next step. But I simply could not do that. Later, someone recommended this acupuncture treatment. I went after work, and the effect was quite obvious.” Here, the choice to continue acupuncture was neither unrestricted nor entirely voluntary; it reflected the limited compatibility of medical recommendations with work, income, and family obligations.

Patients used lived bodily outcomes to compare treatment options, but their decisions remained shaped by economic, familial, and medical conditions. They also relied on other people and digital information when interpreting and comparing those outcomes.

### 4.4 Collective and digital validation

Patients interpreted their bodily experiences through relationships with relatives, neighbors, healthcare workers, other patients, and digital sources. These forms of validation became especially important when relief was partial, symptoms recurred, or patients were uncertain whether bodily change should be attributed to acupuncture. Social confirmation therefore did not merely reinforce an already settled judgment; it helped patients decide how bodily change should be interpreted in the first place.

The gendered composition of the patient sample shaped these processes. Because most patient participants were women, many recommendation pathways moved through female relatives, neighbors, friends, and care networks in which reproductive health, fatigue, body management, chronic pain, and intimate health concerns were discussed with varying degrees of privacy. This does not mean that collective validation was exclusively female; P9's account, for example, also illustrates self-directed therapeutic judgment. Nevertheless, the study's account of social confirmation is shaped by predominantly female patient networks and should not be generalized uncritically to all community acupuncture users.

One common pathway was word-of-mouth recommendation within existing social relationships. Several participants first encountered acupuncture through relatives, neighbors, or acquaintances whose visible improvement made treatment appear plausible. P4 described how a neighbor's recovery from knee discomfort influenced her decision to try acupuncture. The recommendation mattered not simply because advice had been offered, but because the improvement had been witnessed in an everyday social context. The neighbor's visible recovery provided a practical and socially accessible point of comparison before P4 had accumulated her own treatment experience.

Collective validation also occurred within the clinic itself. During one observation, a returning patient rotated her neck after treatment and commented that her movement had become easier. Another patient asked how many sessions she had received, whether the needling was painful, and whether the discomfort returned at night. Through this exchange, bodily change became observable, discussable, and comparable. Therapeutic experience circulated not only through retrospective recommendations, but also through encounters in which patients displayed, questioned, and interpreted one another's bodily responses.

A second pathway involved medically informed endorsement. Some participants were introduced to acupuncture by nurses or acquaintances familiar with both biomedical and Chinese medicine approaches. P2's case is illustrative: a nurse recommended acupuncture on the basis of her own experience and described the practitioners as skillful and attentive. The recommendation carried dual authority because it came from someone personally known to P2 while also drawing legitimacy from proximity to the formal healthcare system. Such endorsement did not remove uncertainty, but it made community-based acupuncture appear sufficiently credible to try.

A third pathway involved digital evaluation, usually in combination with offline recommendation rather than as an independent source of judgment. Before seeking treatment, P7 consulted Meituan to compare price, distance, ratings, and narrative reviews concerning bodily experience, needling sensation, and practitioner technique. P6 similarly used Xiaohongshu to compare accounts of improvement associated with Chinese medicine care with information circulating through friends and local reputation. Digital testimony did not replace interpersonal recommendation; it expanded the range of experiences and practical information available for comparison.

Patients used digital checking to assess whether platform narratives converged with familiar recommendations, local reputation, clinic observation, and their own subsequent bodily responses. Agreement across these sources could reduce uncertainty, whereas inconsistency could introduce doubt. Digital information became persuasive when it aligned with offline relationships and personal experience rather than functioning as a self-sufficient source of authority. In this article, this comparative mechanism is termed convergence testing. Across the cases, interpersonal recommendation, medically informed endorsement, observed interaction, and digital checking made treatment experiences more visible and comparable. However, convergence testing did not guarantee reliability: selective testimony, commercial narratives, and strong local reputation could shape expectations before patients accumulated sufficient personal experience.

### 4.5 Relational trust with safety boundaries

In face-to-face encounters, relational trust was stabilized or limited through practitioner explanation, attentiveness, responsibility, visible safety practices, and recognition of therapeutic and referral boundaries.

P2 emphasized that her consultation involved more than the identification of symptoms such as sleep problems or gastritis. She explained: “The practitioner first took my pulse, then looked at my complexion and my tongue coating. After that, she pointed out symptoms I had recently been having, such as poor sleep, bad breath, and serious hair loss, and asked whether I had a good appetite. Then she also asked whether there was anything troubling me at home. She told me not to put too much pressure on myself, that I was still young, and that my health was the most important thing.” For P2, what mattered was not only the diagnostic explanation, but also the experience of being approached as a whole person.

P3's account shows how explanatory coherence could stabilize trust in the face of medical fear. Suffering from an ovarian cyst and what she described as a “cold womb,” P3 had experienced biomedical care as a sequence of tests, medication, and possible surgery, an option she found frightening. She recalled: “Previously, when I went to the city hospital, the doctor just told me to do tests, then prescribed medicine, or else told me to have surgery. There was no other option. In fact, I was very resistant to surgery, and it made me afraid. But this practitioner kept reassuring me and told me to first focus on regulating my body... Actually, I felt that talking with her made me feel at ease.” For P3, trust was supported not only by the treatment offered, but also by an explanation that made her symptoms feel coherent and manageable.

Relational trust could also fail. In one participant account, a senior Chinese medicine practitioner discussed a patient's gynecological condition in a shaming manner while an assistant was present. The patient experienced the interaction as a violation of dignity and privacy and did not return. This case shows that relational credibility depended not only on attentiveness and explanatory coherence, but also on respectful communication, confidentiality, and moral restraint.

Practitioner accounts complicate a simple opposition between biomedical and community-based care. D1 recognized the value of biomedical examinations: “The advantage of Western medicine is that it is relatively direct. For example, if you have a polyp, whether it is in the gallbladder, stomach, or intestines, examinations can locate it directly.” He contrasted this spatial precision with Chinese medical reasoning, in which the same symptom could have multiple causes.

Practitioners also described referral and the acknowledgment of limits as elements of responsible care. D2 stated: “For serious kidney inflammation, if they come here, we will definitely tell them to go to the hospital first. If they go to the hospital and it does not improve, then they can come back and we can help them regulate it.” D6 similarly emphasized therapeutic limits: “As for a complete cure, that is impossible. Chinese medicine also has its limitations.” These accounts indicate that practitioners framed responsible care not as the unlimited continuation of community-based treatment, but as the recognition of conditions requiring hospital assessment. Such restraint could support relational trust by demonstrating that practitioners acknowledged the limits of their own scope of care.

Visible safety practices also formed part of relational evaluation. Across the observed sites, practitioners used disposable acupuncture needles and alcohol-based disinfection of hands, skin, or reusable equipment. Cups and glass instruments were wiped with alcohol and, in some encounters, exposed to flame. These routines made safety materially visible rather than dependent only on verbal reassurance.

Gloves, masks, and protective clothing were used situationally rather than uniformly, while reusable massage tools and cupping equipment were cleaned between uses. These observations documented the visible cues through which patients could perceive safety; they did not establish infection-control compliance.

Across the cases, trust was strengthened when practitioners explained symptoms, showed attentiveness, used visible safety practices, acknowledged therapeutic limits, and referred patients when necessary. It weakened when patients questioned practitioner technique, responsibility, privacy, or safety.

### 4.6 From perceived benefit to therapeutic credibility

Across the five dimensions, patients repeatedly compared bodily change, durability, everyday fit, social testimony, and practitioner conduct. Perceived benefit contributed to therapeutic credibility when several of these considerations aligned: improvement appeared meaningful, persisted over time, fitted everyday life, received social confirmation, and was accompanied by practitioner responsiveness and visible recognition of therapeutic limits.

These elements did not always reinforce one another. Strong bodily sensations could generate fear, social testimony could amplify expectations, and relational trust could be disrupted by disrespect or failures of privacy. Therapeutic credibility therefore remained provisional and revisable across repeated encounters.

## 5 Discussion

### 5.1 Principal findings

This study conceptualizes therapeutic credibility as an iterative judgment assembled across bodily, temporal, practical, social, and relational domains. Perceived benefit became consequential when several considerations aligned, but credibility remained open to doubt, revision, and discontinuation.

The analysis explains how patient-reported improvement became meaningful enough to guide care within a plural medical setting and how those reports were compared and revised across repeated encounters. The distinction among evidence, evaluation, and credibility is analytically important. Lay therapeutic evidence refers to the grounds patients drew on; therapeutic evaluation refers to the process through which these grounds were compared and revised; and therapeutic credibility refers to the provisional judgment that emerged from that process. Perceived benefit formed one component of lay therapeutic evidence, while credibility also depended on durability, everyday fit, social and digital checking, practitioner responsibility, and perceived safety.

### 5.2 Redefining effective care: medical pluralism as comparative judgment

Patients did not simply reject biomedicine when they entered community-based acupuncture care. They continued to value hospitals for diagnosis, imaging, acute illness, serious disease, and surgery, while turning to acupuncture for chronic discomfort, sleep problems, fatigue, bodily regulation, and concerns they experienced as insufficiently addressed in formal encounters. Their treatment trajectories therefore reflected comparison and combination rather than allegiance to a single medical system.

This finding extends Kleinman's explanatory-model approach by showing that meaningful explanations alone did not sustain care. Patients continued to test explanations against the durability of relief, medication burden, work obligations, practitioner technique, and perceived safety. Explanatory coherence became persuasive when it was accompanied by changes that could be incorporated into everyday life.

The findings also develop Yu's (2021) account of plural healthcare as a process of compatibility and negotiation. Whereas, Yu demonstrates how different medical systems coexist and interact in local society, the present study identifies the evaluative work through which such pluralism is enacted after treatment begins. Patients repeatedly compared bodily responses, recurrence, everyday fit, practitioner conduct, and the possible risks of delayed formal care. Medical pluralism was therefore not only a condition in which several forms of care were available; it was an ongoing practice of therapeutic comparison.

Prolonged ethnographic engagement made visible repeated shifts between confidence and doubt that a single interview or attitude measure might present as a stable treatment preference. What appeared to be a choice for acupuncture was often a continuing judgment about which forms of care remained tolerable, accessible, and compatible with work and family responsibilities.

### 5.3 Embodied and temporal testing of therapeutic evidence

Building on Csordas's (1990) account of the body as the ground of perception and Pritzker's (2011) analysis of how Chinese medical sensations are interpreted through therapeutic discourse, the findings show that bodily experience was not settled at the moment of sensation. Its significance emerged through remembered baselines, recurrence, daily functioning, and later comparison.

Patients did not merely report pain relief, soreness, warmth, numbness, or relaxation. They monitored how long changes lasted, whether familiar symptoms returned, whether flare-ups became less frequent, and whether improvement carried into work, sleep, movement, and care responsibilities. Immediate bodily difference therefore became meaningful through what the findings term temporal testing.

This temporal dimension also preserved the ambiguity of bodily experience. A strong needling sensation could be interpreted as therapeutic activation, excessive stimulation, or possible injury. Practitioner explanation could make the experience more intelligible, but it did not necessarily eliminate doubt. Bodily sensation was therefore neither self-evident proof nor a fixed satisfaction response; it was material for continuing interpretation.

The findings refine embodiment-oriented approaches by showing that the body was not only where illness and treatment were experienced, but also where competing therapeutic claims were repeatedly tested. Bodily memory, recurrence, and practical continuity became evaluative resources through which patients compared present change with previous treatment and familiar patterns of suffering.

Formal outcome records were not consistently available in the observed community settings. Patients consequently relied on memory, symptom recurrence, everyday functioning, and repeated self-comparison. Such judgments may correspond partly to changes captured by clinical measures, but they may also be influenced by symptom fluctuation, concurrent care, expectations, and interpretive framing. Their sociological significance lies in how they functioned as situated forms of lay therapeutic reasoning and shaped whether treatment remained worth pursuing.

### 5.4 Therapeutic agency under constraint

The findings also extend medical-sociological accounts of agency in chronic illness management (Bury, 1982; Charmaz, 1983; Mol, 2008). Patients did not simply comply with professional advice or reject medical authority. They compared recommendations with medication side effects, recurring symptoms, work demands, family obligations, and their own accumulated bodily experiences.

Agency appeared less as sovereign choice than as the capacity to compare and adjust care under constrained conditions. Patients could question a particular recommendation or continue acupuncture while still relying on hospital examinations, prescribed medication, family advice, and professional monitoring. Choosing community-based acupuncture therefore did not necessarily involve replacing one system of expertise with another.

This interpretation is consistent with the Burden of Treatment Theory, which emphasizes that patients' capacity to manage care is shaped by the practical work imposed by illness and treatment (May et al., 2014). In the present study, wage labor, caregiving, cost, travel, scheduling, and concern about delayed diagnosis all shaped what patients could realistically do. Treatment decisions were therefore made within unequal material and relational conditions.

The contribution is to locate therapeutic agency not only in the moment of choosing a treatment, but in the continuing work of comparing consequences. Patients assessed what enabled them to function, what produced tolerable burdens, and what required further medical monitoring. Agency was thus negotiated through repeated adjustment rather than expressed as either unrestricted autonomy or passive compliance.

### 5.5 Collective and digital validation: socially distributed therapeutic interpretation

Patients' interpretations of treatment were not produced individually. Relatives, neighbors, nurses, other patients, practitioners, and digital platforms all contributed to how bodily change became recognizable and credible. Social confirmation was particularly important when relief was partial, symptoms recurred, or patients were uncertain whether change should be attributed to acupuncture.

This finding develops health-literacy research, which commonly emphasizes an individual's capacity to access, understand, and evaluate health information (Nutbeam, 2000; Bains and Egede, 2011; Sørensen et al., 2012; Okawa et al., 2023). In the observed settings, evaluation was socially distributed. Patients learned how to interpret treatment through familiar recommendations, witnessed improvement, medically informed endorsement, conversations in waiting areas, practitioner explanations, and their own later bodily responses.

Digital information was rarely treated as an independent source of authority. Patients assessed platform reviews alongside familiar recommendations, local reputation, clinic observation, and personal experience. This finding extends Chinese digital-health research on credibility assessment and online reputation signals (Deng et al., 2019; Chang et al., 2024) by showing how digital information became meaningful in community-based care.

The comparative mechanism defined in the findings as convergence testing involved cross-checking platform narratives, interpersonal testimony, observed practice, and subsequent bodily experience. Agreement across these sources could reduce uncertainty, whereas inconsistency could introduce doubt. Digital platforms therefore expanded the range of information available for comparison, but they did not replace socially embedded judgment.

Convergence did not guarantee reliability. Selective success stories, commercial promotion, and strong local reputation could mutually reinforce expectations before patients' accumulated sufficient personal experience. Collective validation could therefore support therapeutic navigation while also amplifying exaggeration or making persuasive testimony appear more reliable than it was.

Because most patient participants were women, many of these recommendation pathways operated through predominantly female care networks. The pattern should therefore be interpreted as gendered and context-specific rather than generalized to all community acupuncture users.

### 5.6 Relational trust with safety boundaries

Trust emerged as both a relational and an institutional problem. Sociological research has shown that trust in healthcare is shaped by competence, communication, care, vulnerability, and perceived responsibility rather than by interpersonal liking alone (Mechanic and Meyer, 2000; Hall et al., 2001; Gilson, 2003). The present findings show how these processes operate where formal institutional guarantees are uneven.

In formal healthcare settings, authority is supported by professional licensing, organizational accountability, diagnostic technologies, and standardized procedures. In the community-based settings observed here, authority was assembled through a different combination of resources: practical experience, local reputation, perceived technical skill, patient testimony, careful explanation, attentiveness, and moral restraint. These resources help explain why patients could experience community-based care as credible, but they were not equivalent to formal medical authority or regulatory oversight.

Practitioner explanation was particularly important because bodily sensations did not interpret themselves. By connecting symptoms with work, stress, diet, sleep, and bodily constitution, practitioners could make suffering feel coherent and manageable. Recognition of biomedical examinations, acknowledgment of therapeutic limits, and referral for serious conditions further strengthened credibility by demonstrating restraint rather than unlimited therapeutic claims.

Relational familiarity, however, was not inherently protective. The negative account in which a patient's gynecological condition was discussed in a shaming manner illustrates how trust could fail through violations of dignity and privacy. Familiarity and local reputation could also reduce patients' willingness to question technique, credentials, or the need for biomedical assessment. Trust therefore depended not only on warmth and explanatory coherence, but also on respectful communication, confidentiality, and recognition of professional limits.

Visible hygiene practices made safety materially perceptible. Disposable needles, alcohol-based disinfection, and the cleaning of reusable tools communicated attentiveness and responsibility. As specified in the Methods, these observations concerned visible safety cues rather than verified infection-control compliance.

Relational trust should therefore not be treated as a substitute for formal safeguards. It became more accountable when attentiveness and explanation were accompanied by transparent safety practices, communication of uncertainty, acknowledgment of therapeutic limits, and appropriate referral. In informal and semi-formal community settings, trust could support safer navigation only when it remained open to questioning and was bounded by responsibility.

### 5.7 Theoretical and practical contributions

This study makes three contributions.

First, it conceptualizes therapeutic credibility as a recursive and provisional judgment rather than a fixed belief, stable attitude, or one-time treatment decision. Perceived benefit became consequential through repeated comparison, while therapeutic credibility remained subject to doubt, revision, and discontinuation.

Second, it shows how bodily comparison, practical constraints, social and digital checking, and practitioner conduct interact after patients enter care. Medical pluralism was therefore enacted not only through treatment choice, but through continuing evaluative work.

Third, the study shows that relational trust becomes accountable only when explanation and attentiveness are accompanied by visible safety practices, recognition of therapeutic limits, respect for privacy and dignity, and appropriate referral. Trust could support continued care, but it could not substitute for diagnostic monitoring, regulation, or formal safeguards.

These findings also have practical implications. Communication should begin with patients' experiential reasoning rather than with abstract correction. Formal healthcare providers can ask what changes patients noticed, how long they lasted, what other treatments were being used, who recommended the practice, and whether symptoms required continued diagnostic monitoring. Community practitioners can support safer navigation by avoiding guaranteed claims, communicating uncertainty, maintaining transparent hygiene practices, respecting confidentiality, and referring severe, persistent, or unfamiliar conditions for formal assessment.

### 5.8 Limitations and future research

Several limitations delimit the claims. First, the study mainly included patients who had completed at least three treatment sessions and therefore overrepresents continued engagement. Patients who discontinued treatment, reported dissatisfaction, experienced possible adverse effects, or distrusted practitioners were not systematically recruited. Their accounts may reveal how therapeutic credibility fails, weakens, or is retrospectively revised.

Second, the sample was small, context-specific, and strongly gendered: 10 of the 11 patient participants were women. The findings may consequently overrepresent recommendation pathways, intimate-health concerns, and relational evaluations circulating through women's care networks. Gatekeeper and snowball recruitment may also have favored participants with more established or positive practitioner relationships. Routes of field access may have shaped which participants felt comfortable discussing reproductive concerns, bodily symptoms, or emotional distress.

Third, the study did not collect standardized clinical outcome measures and therefore cannot estimate the extent to which participants' reported improvements corresponded to changes in clinical indicators. It also did not conduct a systematic infection-control audit. Fieldnotes documented visible practices but did not assess sterilization records, waste disposal, technical compliance, or microbiological safety.

Fourth, practitioner perspectives were used primarily to contextualize patient evaluation rather than analyzed as an independent object of inquiry. A practitioner-centered ethnography could examine more fully how community-based providers understand authority, responsibility, uncertainty, scope of practice, and referral.

Future research should recruit patients at treatment initiation and follow trajectories of improvement, doubt, discontinuation, adverse experience, referral, and return to formal healthcare. Comparative research could examine how hospital-based and community-based settings differently organize therapeutic evaluation, trust, and safety-related decision-making. Mixed-methods studies could further clarify how patient-reported interpretations relate to clinical indicators, safety events, and longer-term care pathways.

## 6 Conclusion

This study shows that patients evaluated community-based acupuncture care in southwest China by comparing bodily change over time, weighing treatment against everyday constraints, consulting social and digital sources, and assessing practitioner responsibility and safety. Through repeated comparison, interpretation, and discussion, these experiences became lay therapeutic evidence that shaped whether care remained credible enough to continue.

The study contributes to medical sociology by conceptualizing therapeutic credibility as a conditional judgment assembled and revised through continued care. Perceived benefit became consequential when improvement appeared durable, fitted everyday life, received social confirmation, and was accompanied by practitioner responsiveness and recognition of therapeutic limits. Understanding this process can support communication that takes patient experience seriously while maintaining attention to uncertainty, diagnostic monitoring, respectful care, appropriate referral, and scope-of-practice boundaries.

## Data Availability

The datasets presented in this article are not readily available because the dataset contains sensitive interview and ethnographic materials. To protect participant confidentiality, the data are not publicly available. De-identified data may be available from the authors upon reasonable request and subject to ethical restrictions. Requests to access the datasets should be directed to xu1991122@gmail.com.
