# Supplementary material for: Therapeutic credibility in community-based acupuncture care: an ethnographic study in southwest China
Source: Front Sociol. 2026 Aug 26;11:1908107. doi: 10.3389/fsoc.2026.1908107 (PMC13553223; doi:10.3389/fsoc.2026.1908107)
Supplement: Supplementary file 1 [file Table_1.docx]

# **Supplementary Methods Tables**

**Supplementary Table S1. Field sites and observation contexts**

| **Site** | **Setting and formality** | **Observation focus** | **Hours** | **Services** |
| --- | --- | --- | --- | --- |
| Site A | Residential family-based clinic; home-based / informal | Referrals; domestic-clinical space; repeated return | about 90 | Acupuncture; tuina massage; private wellness care |
| Site B | Center near a TCM hospital; semi-clinical | Institutional-community boundary; diagnosis and regulatory adaptation | about 80 | Acupuncture; moxibustion; tuina; cupping |
| Site C | Border-county rehabilitation space; semi-formal | Patient mobility; access; hybrid knowledge | about 65 | Acupuncture; moxibustion; tuina; cupping |
| Site D | Community acupuncture center; informal / semi-formal | Local reputation; practitioner-patient familiarity | about 70 | Herbal preparations; acupuncture; moxibustion |

*Note.* Site names and identifying locations are generalized to protect participants and practitioners.

**Supplementary Table S2. Patient participants**

| **ID** | **Gender / age** | **Main concern** | **Exposure / interviews** | **Evaluative context** | **Site / entry** |
| --- | --- | --- | --- | --- | --- |
| P1 | Female / 36 | Lumbar ligament injury; neck and shoulder discomfort | 12 sessions; ~2 months; 2 interviews | Patches and painkillers; medication burden, durability, strong-needling doubt | A; colleague |
| P2 | Female / 33 | Sleep disorder; fatigue; gastritis | 12 sessions; ~2 months; 2 interviews | Whole-person explanation, reassurance, regulatory ambiguity | A; medically informed acquaintance |
| P3 | Female / 30 | Ovarian cyst; cold-womb symptoms; gastritis | 6 sessions; ~1.5 months; 2 interviews | Hospital tests, medication, possible surgery; reassurance and safety boundary | B; friend |
| P4 | Female / 27 | Arthritis | 6 sessions; ~1.5 months; 1 interview | Hospital and TCM care; work constraint and technique-dependent benefit | B; friend |
| P5 | Female / 35 | Lumbar muscle strain | Ongoing ~3 months; 1 interview | Patches and painkillers; childbirth and work burden | A; family |
| P6 | Female / 29 | Gastrointestinal issues; allergy | 12 sessions; ~2 months; 1 interview | Digital checking, preference for Chinese medicine, feedback-based adjustment | B; digital search |
| P7 | Female / 24 | Upper-back or neck prominence, described as a dowager's hump | 8 sessions; ~2 months; 1 interview | Meituan comparison, visible change, trust around payment | C; Meituan |
| P8 | Female / 32 | Gynecological issues | Ongoing ~2 months; 1 interview | Hospital and medication experience; triage and intimate health | C; friend |
| P9 | Male / 40 | Cervical spondylosis; headache; sleep disorder; joint effusion | Ongoing ~3 months; 2 interviews | Medication side effects, professional advice, self-directed comparison | C; friend |
| P10 | Female / 55 | Diabetes; hypertension | Ongoing ~2 years; 1 interview | Biomedical monitoring; measured versus felt body | D; friend |
| P11 | Female / 45 | Weight loss; cosmetic treatment | Ongoing ~1 year; 2 interviews | Body management, maintenance, expectations | D; family |

*Note.* Identifying occupational and location details are generalized or omitted. Treatment exposure and evaluative context summarize information relevant to the analysis rather than complete medical histories.

**Supplementary Table S3. Practitioner participants**

| **ID** | **Gender / age** | **Context and services** | **Experience / interviews** | **Training / credentialing** |
| --- | --- | --- | --- | --- |
| D1 | Male / 43 | Community and lineage practice; acupuncture, moxibustion, herbal prescriptions | 20 years; 2 interviews | Lineage training and mentorship; formal credentialing details not reported |
| D2 | Female / 45 | Health-cultivation and community care; acupuncture, moxibustion, massage, cupping, intimate health | 11 years; 4 interviews | Nursing background and mentorship-based acupuncture training |
| D3 | Female / 58 | Family-lineage and local practice; acupuncture, moxibustion, herbal prescriptions | 15 years; 2 interviews | TCM medical training and mentorship-based practice |
| D4 | Female / 42 | Community care; acupuncture, moxibustion, massage | 13 years; 2 interviews | Medical acupuncture training combined with paternal lineage training |
| D5 | Female / 51 | Community care; acupuncture, moxibustion, massage, cupping | 20 years; 2 interviews | Long practical experience and local reputation; details generalized |
| D6 | Male / 27 | Community acupuncture; acupuncture and moxibustion | 5 years; 1 interview | Community-based experience; physician qualification discussed as an authority boundary |

*Note.* Training and credentialing details are generalized because several sites occupied semi-formal or community-based positions. The table does not constitute a legal assessment of certification status.
